# Long‐term follow‐up of fenestrated endovascular repair for juxtarenal aortic aneurysm

**DOI:** 10.1002/bjs.10524

**Published:** 2017-04-12

**Authors:** I. N. Roy, A. M. Millen, S. M. Jones, S. R. Vallabhaneni, J. R. H. Scurr, R. G. McWilliams, J. A. Brennan, R. K. Fisher

**Affiliations:** ^1^ Liverpool Vascular and Endovascular ServiceRoyal Liverpool University HospitalLiverpoolUK; ^2^ Interventional RadiologyRoyal Liverpool University HospitalLiverpoolUK; ^3^ Institute of Ageing and Chronic DiseaseUniversity of LiverpoolLiverpoolUK

## Abstract

**Background:**

Fenestrated endovascular aneurysm repair (FEVAR) is increasingly being used for juxtarenal aortic aneurysms. The aim of this study was to review long‐term results and assess the importance of changing stent‐graft design on outcomes.

**Methods:**

This was a retrospective review of all patients who underwent FEVAR within a single unit over 12 years (February 2003 to December 2015). Kaplan–Meier analysis of survival, and freedom from target vessel loss, aneurysm expansion, graft‐related endoleak and secondary intervention was performed. Comparison between outcomes of less complex grafts (fewer than 3 fenestrations) and more complex grafts (3 or 4 fenestrations) was undertaken.

**Results:**

Some 173 patients underwent FEVAR; median age was 76 (i.q.r. 70–79) years and 90·2 per cent were men. Median aneurysm diameter was 63 (59–71) mm and median follow‐up was 34 (16–50) months. The adjusted primary technical operative success rate was 95·4 per cent. The in‐hospital mortality rate was 5·2 per cent; there was no known aneurysm‐related death during follow‐up. Median survival was 7·1 (95 per cent c.i. 5·2 to 8·1) years and overall survival was 60·1 per cent (104 of 173). There was a trend towards an increasing number of fenestrations in the graft design over time. In‐hospital mortality appeared higher when more complex stent‐grafts were used (8 versus 2 per cent for stent‐grafts with 3–4 versus fewer than 3 fenestrations; P = 0·059). Graft‐related endoleaks were more common following deployment of stent‐grafts with three or four fenestrations (12 of 90 versus 6 of 83; P < 0·001).

**Conclusion:**

Fenestrated endovascular aneurysm repair for juxtarenal aneurysm is associated with few aneurysm‐related deaths in the long term. Significant numbers of secondary interventions are required, but the majority of these can be performed using an endovascular approach.

## Abstract 2

Prevents aneurysm rupture

## Introduction

The treatment of a juxtarenal aortic aneurysm by fenestrated endovascular aneurysm repair (FEVAR) was first reported in 1999^1^. FEVAR has become the commonest treatment for juxtarenal aneurysms, with 263 procedures reported in the UK in 2015^2^. The UK‐wide BSET GLOBALSTAR (British Society for Endovascular Therapy: Global Collaborators on Advanced Stent‐Graft Techniques for Aneurysm Repair) registry has established the safety and efficacy of the technique by reporting short‐ and medium‐term outcomes of initial procedures undertaken during 2003–2009^3^. Reports of long‐term outcomes are sparse^4^ and as such long‐term durability remains uncertain.

FEVAR stent‐graft design has changed over the past decade, with a trend towards a more proximal seal zone, necessitating more fenestrations or scallops to maintain visceral perfusion. Although this may allow the treatment of more complex aneurysms, it is perceived by the authors that it is also being applied to juxtarenal aneurysms that were previously treated using less complex grafts.

BSET GLOBALSTAR^3^ recorded an increased inpatient mortality rate of 9·4 per cent after use of stent‐grafts that incorporated the coeliac trunk compared with 2·8 per cent for those that did not. As such, the widely reported short‐ and medium‐term outcomes of early case series may not reflect the outcomes of stent‐grafts used in modern practice.

The aim of this study was to assess the overall long‐term outcomes following FEVAR for the treatment of juxtarenal aortic aneurysms in a single centre, and to observe the effect of changes in stent‐graft design.

## Methods

Data for all patients undergoing FEVAR for juxtarenal aortic aneurysm in the Liverpool Vascular and Endovascular Service between February 2003 and December 2015 were included in the analysis. Patients who underwent an endovascular repair involving branched components or fenestrations with a proximal thoracic extension were excluded as these procedures were deemed to represent thoracoabdominal aneurysm repairs. Information was obtained by retrospective review of institutional data including patient demographics, aneurysm characteristics, operative details and postoperative surveillance. Confirmation of mortality data were obtained from National Health Service (NHS) institutional information. Cause of death was obtained from patient's general practitioners or death certificates.

A target vessel was defined according to a previously published report^5^ on FEVAR, as ‘a vessel potentially covered by the stent‐graft fabric if not for a deliberate mechanism of preservation, when the stent‐graft is deployed as intended’. A fenestration was defined as a ‘deliberate defect either circular or elliptical’ and a scallop as a ‘U‐shaped gap in the proximal fabric of the graft’^5^.

Two subgroups were created, divided by the median number of fenestrations in implanted stent‐grafts. This created a group of grafts with no, one or two (fewer than 3) fenestrations and a group with three or four fenestrations. Clinically, this relates to patients who would normally undergo stenting of a mesenteric vessel (3 or 4 fenestrations) or not (fewer than 3).

The primary endpoint was overall survival. Secondary endpoints were freedom from target vessel loss, aneurysm growth, graft‐related (type I or III) endoleak and secondary intervention. Definitions of success, complications and other events associated with endovascular repair were in accordance with accepted reporting standards^6^, ^7^. The primary outcome was calculated using NHS data and as such patients were censored only at the point of data collection. If the patient was known to be alive and still on the surveillance programme, their last follow‐up surveillance imaging was taken as the last point of data collection for secondary outcomes. If the patient was followed up elsewhere and hence not in the local programme, their last point of imaging was taken as the last follow‐up time point for secondary outcomes. All planned secondary interventions were undertaken within the primary institution; unplanned interventions were detected on the next imaging or recorded if correspondence was received. This arrangement was limited to the author's early experience as all patients now attend this institution for follow‐up imaging.

### Assessment of renal function

Preoperative renal function was calculated as creatinine clearance, using the Cockcroft–Gault formula. All calculations were based on ideal bodyweight calculated from the patient's sex and preoperative height.

### Follow‐up protocol

A standardized post‐FEVAR surveillance protocol was developed including plain abdominal X‐ray before discharge, duplex ultrasonography and single arterial‐phase CT angiography (CTA) after 1 month, with clinical review 6 weeks after surgery. Abdominal X‐ray, duplex ultrasonography and CTA were repeated after 6 months, then annually. If complications or potential problems were identified, patients were discussed at a multidisciplinary team meeting. Further imaging in the form of triple‐phase CTA or contrast‐enhanced ultrasonography was performed if deemed appropriate, and secondary intervention was undertaken if indicated clinically. Loss of a target vessel was defined as complete occlusion of the target vessel main stem.

### Statistical analysis

Continuous data are presented as median (i.q.r.). Overall survival, target vessel loss, graft‐related endoleak and secondary intervention were all subject to Kaplan–Meier analysis and log rank comparison using RStudio® version 0.99 (RStudio, Boston, Massachussetts, USA). Median follow‐up was determined by means of the reverse Kaplan–Meier technique. All other statistical analysis was done using SPSS® version 22 (IBM, Armonk, New York, USA).

## Results

During the 12‐year study interval, some 209 patients underwent a branched and/or fenestrated procedure in the authors' institution, of whom 174 were eligible for inclusion in this analysis. One patient in whom the operation failed was excluded from the analysis. The device could not be introduced past a stenotic aortic bifurcation and the procedure was abandoned. This patient remained alive 34 months after the failed operation. Baseline data for the remaining 173 patients are summarized in *Table* 1.

**Table 1:** Preoperative data for patients undergoing fenestrated endovascular repair in a single UK centre

	No. of patients* (*n* = 173)
Age (years)†	76 (70–79)
Sex ratio (M : F)	156 : 17
BMI (kg/m^2^)†	27·4 (25·0–30·0)
Diabetic	
Yes	28 (16·2)
No	145 (83·8)
Ischaemic heart disease	
Known	91 (52·6)
Not known	82 (47·4)
Hypertension	
Known	110 (63·6)
Not known	63 (36·4)
Smoking status	
Smoker	31 (17·9)
Ex‐smoker	110 (63·6)
Non‐smoker	32 (18·5)
Previous aortic surgery‡	*n* = 103
Yes	9 (8·7)
No	94 (91·3)
ASA fitness grade	*n* = 168
I	2 (1·2)
II	49 (29·2)
III	112 (66·7)
IV	5 (3·0)
Haemoglobin (g/l)†	138 (127–147)
Systolic BP (mmHg)†	135 (122–146)
Preoperative ECG	*n* = 162
Normal	76 (46·9)
Atrial fibrillation	12 (7·4)
Other abnormality	74 (45·7)
Left ventricular ejection fraction (%)†	60 (51–64)
FEV1 (litres)†	2·1 (1·6–2·6)
Creatine clearance (ml/min)†	55 (43–65)
Chronic kidney disease stage	*n* = 169
I	3 (1·8)
II	66 (39·1)
III	84 (49·7)
IV	15 (8·9)
Dialysis	1 (0·6)
Aneurysm diameter (mm)†	63 (59–71)

* With percentages in parentheses unless indicated otherwise;

† values are median (i.q.r.).

‡ Fenestrated endovascular repair to treat complication. FEV1, forced expiratory volume in 1 s.

### Stent‐graft configuration

**Figure 1:**
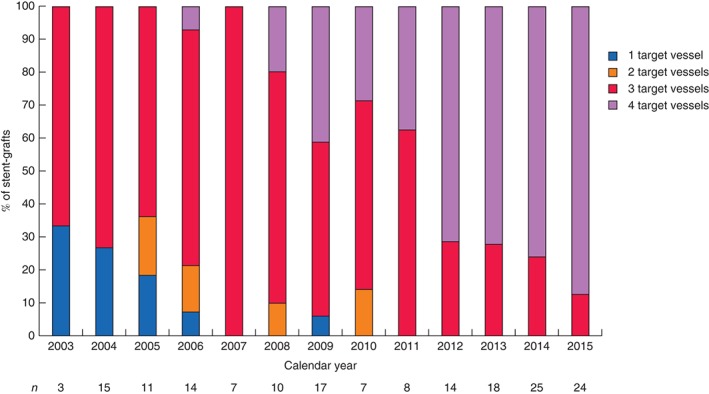
Percentage of stent‐grafts with each number of target vessels by calendar year

All patients were treated with fenestrated stent‐grafts produced by a single manufacturer (Cook Medical, Bloomington, Indiana, USA). The stent‐graft configuration was recorded for all patients (*Table* 2). The most common stent‐graft and target vessel configuration was a scallop for the superior mesenteric artery (SMA) and a fenestration for each of the renal arteries (62 patients, 35·8 per cent). Over the duration of the study there was a trend towards an increasing number of target vessels (*Fig*.  1). Comparison of 2004 and 2015 showed a significant difference in the number of target vessels (median 3 *versus* 4; *Z*‐score = 4·64, *P* < 0·001, Mann–Whitney *U* test). Aneurysm and stent‐graft D1 (neck) diameter did not display any trend over time and are presented as surrogate markers of aneurysm morphology (*Figs S1* and *S2*, supporting information).

**Table 2:** Fenestrated endovascular aneurysm repair stent‐graft configuration in a single UK centre

No. of target vessels	No. of patients	Coeliac axis	SMA	RRA	LRA
4 (44·5)	30	Fenestration	Fenestration	Fenestration	Fenestration
	47	Scallop	Fenestration	Fenestration	Fenestration
3 (46·8)	11		Fenestration	Fenestration	Fenestration
	62		Scallop	Fenestration	Fenestration
	4	Fenestration	Fenestration	Fenestration to RRA or LRA renal	
	3	Scallop	Fenestration	Fenestration to RRA or LRA renal	
	1		Scallop	Scallop	Fenestration
2 (3·5)	1*	Fenestration	Fenestration		
	1		Fenestration	Fenestration	
	1		Scallop	Fenestration	
	1			Fenestration	Fenestration
	2			Fenestration	Scallop
1 (5·2)	9			Scallop to RRA or LRA renal	

Values in parentheses are percentage of patients.

* Patient with end‐stage renal failure on dialysis. SMA, superior mesenteric artery; RRA, right renal artery; LRA, left renal artery.

### Operative data

The fenestrated device was introduced successfully in 173 patients. Two target vessels (0·3 per cent) were lost during surgery, in separate patients; both were renal arteries. There were 35 patients with a graft‐related endoleak (type I or III) on completion of the procedure. The primary technical success rate was 79·2 per cent (137 of 173 patients). Twenty‐nine of the 35 graft‐related endoleaks identified on completion angiography had resolved without intervention by the 1‐month surveillance imaging. This gave an adjusted primary technical success rate of 95·4 per cent (165 patients).

The median duration of operation was significantly shorter for stent‐grafts implanted with fewer than three fenestrations than those with three or four fenestrations (285 (95 per cent c.i. 240 to 330) versus 360 (300 to 485) min; P < 0·001). Some 29 unplanned intraoperative manoeuvres were required to ensure adequate aneurysm treatment in 29 patients (Table 3).

**Table 3:** Unplanned intraoperative manoeuvres

	Reason	No. of patients
Extra target vessel stent	Maldeployment/endoleak	5
	Target vessel dissection	2
	Target vessel perforation	2
	Unknown	2
Unplanned upper limb access	Failure to cannulate target vessel	2
Limb extension/Wallstent™	Kink/flow limitation	7
	Type Ib endoleak	3
	Iliac rupture	2
	Insufficient limb overlap	1
Unplanned femorofemoral bypass	Insufficient limb flow	2
Unplanned iliofemoral bypass	Iliac rupture	1

Wallstent™ **(**Boston Scientific, Marlborough, Massachusetts, USA).

### Target vessels

There was a total of 572 target vessels. Of these, 126 (22·0 per cent) were preserved with a scallop and 446 (78·0 per cent) with a fenestration. It was routine practice to place a stent in all fenestrations; however, this was not possible in four instances.

Of the 126 vessels preserved with a scallop, ten (7·9 per cent) required a stent (9 renal arteries and 1 SMA). In total 452 vessels were stented. The stent type was known in 419 procedures. Bare metal stents were used in 80 target vessels (53 Palmaz® Genesis®, Cordis, Miami Lakes, Florida, USA; 27 others). Covered stents were used in 339 target vessels (325 Advanta™, Atrium Medical, Hudson, New Hampshire, USA; 14 others).

### Perioperative mortality and complications

A total of nine patients (5·2 per cent) died during the primary hospital admission. The cause of death was embolic ischaemia of abdominal viscera (3), myocardial infarction (2), multiple organ failure (2), retroperitoneal bleed from a target vessel (1) and aspiration pneumonia (1). Of those who died from abdominal viscera ischaemia, one patient had a stent‐graft with four fenestrations, and the remaining two patients had stent‐grafts with three fenestrations and a scallop for the coeliac axis.

Mortality rates according to stent‐graft complexity were similar: 2 of 83 patients (2 per cent) who received devices with fewer than three fenestrations died in hospital, compared with seven of 90 (8 per cent) whose stent‐grafts had three or four fenestrations (P = 0·059).

In the postoperative period, 47 patients (27·2 per cent) had 53 primary complications which prolonged hospital stay (Table 4). Both patients with transient paraplegia underwent implantation of four‐fenestration stent‐grafts and had patent lumbar arteries excluded by the stent‐graft. One had planned operative occlusion of a single internal iliac artery. Both had spinal drains inserted on discovery of their symptoms and subsequently recovered. Eleven patients required a surgical intervention. These are distinct from the five patients who had secondary interventions to maintain FEVAR efficacy before discharge, reported in secondary interventions.

**Table 4:** Inpatient complications prolonging hospital stay and surgical interventions

	Complication	No. of patients	Surgical interventions
Cardiac	Acute coronary syndrome	8	
	Cardiac failure	4	
	Symptomatic arrhythmia	4	
Respiratory	Pneumonia	8	
	Acute respiratory distress syndrome	1	
Neurological	Acute delirium	3	
	Transient paraplegia	2	Spinal drainage
Urinary	Acute kidney injury	7	Temporary dialysis 3
	Acute retention	2	
	Urinary tract infection	2	
	Renal hypertension from ischaemia	1	
Gastrointestinal	Bleeding	1	Upper gastrointestinal endoscopy
	Ischaemia	4	Gastrectomy and splenectomy 1* Left hemicolectomy 1† Hartmann's procedure 1* Acute SMA stent angioplasty 1†
	Prolonged ileus	2	
Access complications	Groin bleeding	3	Surgical exploration
	Bypass graft occlusion	1	Redo iliofemoral graft

* Died from complication.

† Stent‐graft contained four fenestrations. SMA, superior mesenteric artery.

Median length of hospital stay was 7 (5–11) days (167 patients), and did not differ between stent‐graft complexity groups (P = 0·476).

### Renal function

Three patients (1·7 per cent) required temporary dialysis, but none needed new permanent dialysis (Table 4). Forty‐seven (27·8 per cent) of 169 patients with available data suffered from acute kidney injury (more than 1·5‐fold increase in serum creatinine). No patient required new permanent dialysis in recorded follow‐up.

### Follow‐up

**Figure 2:**
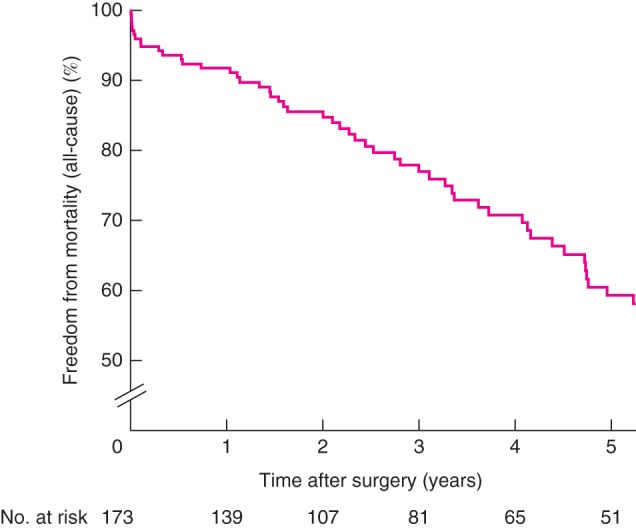
Freedom from mortality (all‐cause) following fenestrated endovascular aneurysm repair in a single UK centre

Median follow‐up was 34 (i.q.r. 16–50) months. All‐cause survival was 59·4 per cent (95 per cent c.i. 50·7 to 69·5) years at 5 years. Overall median survival was 7·1 (95 per cent c.i. 5·2 to 8·1) years. Sixty‐nine patients died during follow‐up (Fig. 2). There were no reported aneurysm‐related deaths. Freedom from loss of any target vessel and secondary intervention was 90·1 (82·9 to 97·9) and 62·8 (51·7 to 76·3 per cent) respectively at 5 years. Ten‐year results for each outcome are presented in Figs S3–S7 (supporting information).

**Figure 3:**
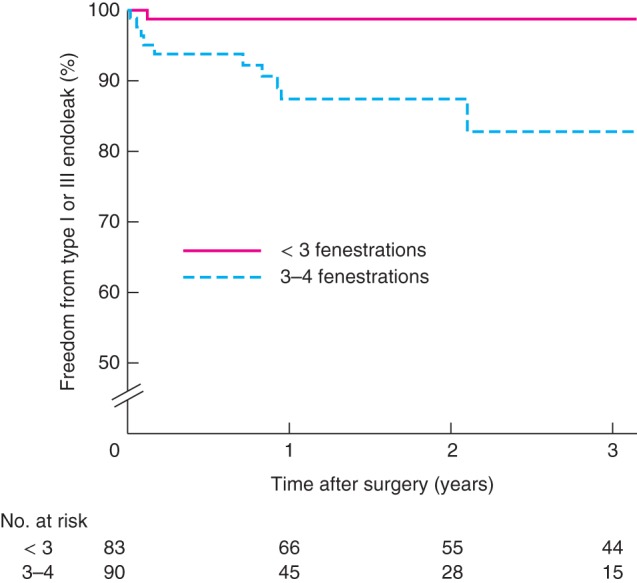
Freedom from type I or III endoleak following fenestrated endovascular aneurysm repair in a single UK centre in relation to number of fenestrations in stent‐graft. P < 0·001 (log rank test)

**Figure 4:**
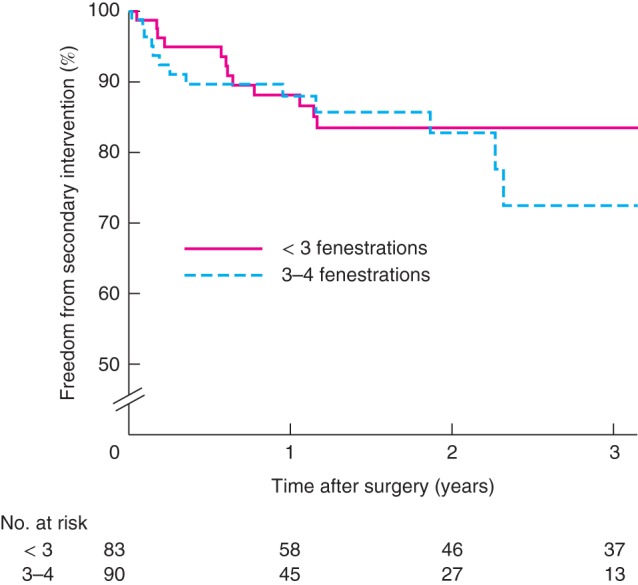
Freedom from secondary intervention following fenestrated endovascular aneurysm repair in a single UK centre in relation to number of fenestrations in stent‐graft. P = 0·508 (log rank test)

Rates of graft‐related endoleak were higher in patients who received stent‐grafts with three or four fenestrations *versus* fewer than three fenestrations (12 of 90 (13 per cent) *versus* 6 of 83 (7 per cent) respectively; *P* < 0·001) (*Fig*. 3). There were no significant differences between groups in secondary interventions (20 of 90 (22 per cent) *versus* 14 of 83 (17 per cent); *P =* 0·508) (*Fig*.  4), aneurysm growth (18 of 90 (20 per cent) *versus* 19 of 83 (23 per cent); *P* = 0·160) (*Fig. S8*, supporting information) and freedom from loss of a target vessel (84 of 90 (93 per cent) *versus* 78 of 83 (94 per cent); *P =* 0·272) (*Fig. S9*, supporting information). Survival was similar in the groups with more and less complex stent‐grafts (72 of 90 (80 per cent) *versus* 32 of 83 (39 per cent); *P =* 0·264) (*Fig. S10*, supporting information),

Thirty‐four patients required a secondary intervention during follow‐up. The first secondary intervention was endovascular in 30 patients and four required open surgery. Most interventions were on target vessel stents (19) or limbs (8), and were indicated by endoleaks or stenosis. Six patients required more than one secondary intervention, which resulted in a total endovascular rate of 82 per cent (36 of 44) for all secondary interventions.

## Discussion

FEVAR is an effective and safe method for treating aneurysms not suitable for standard endovascular repair, in the short to medium term. These observations were confirmed in the present cohort. The inpatient mortality rate of 5·2 per cent in this series is higher than the GLOBALSTAR rate of 4·1 per cent^4^. Meta‐analyses have reported a pooled mortality rate of 2 per cent^8^ and 2·5 per cent^9^ within 30 days, lower than the 30‐day mortality rate of 3·4 per cent the present cohort. One possible explanation for this is the use of a higher proportion of devices with three or four fenestrations in the present series.

One of the difficulties associated with reporting of outcomes of patients with aortic aneurysms is the lack of standard reporting criteria. No clear guidance exists to define juxtarenal aneurysm. A number of terms are used in everyday clinical practice, including juxtarenal, pararenal and suprarenal aneurysm. It would help greatly in comparing the outcomes of different interventions if these were defined consistently. The present series demonstrated a trend towards higher hospital mortality after deployment of more complex stent‐grafts (3 or 4 fenestrations) and this may simply be a reflection of the more complex aortic morphology. Over time there has been an increasing use of more complex stent‐grafts to deal with juxtarenal aneurysms in the author's centre, and anecdotally among other centres. The advantage of using more complex stent‐grafts is that the sealing zone is generally pushed higher in the aorta, thus offering a potentially more durable proximal seal in relatively healthy aorta. More complex stent‐grafts may come at a cost of increasing operating time and rates of graft‐related endoleak because of the larger number of fenestrations. The lack of difference in rates of aneurysm expansion, secondary interventions and all‐cause mortality between stent‐grafts of varying complexity may simply be due to the relatively small numbers of patients.

The primary technical success rate in this series of 79·2 per cent on completion angiography was relatively lower than in other contemporary series (96·8 per cent)^8^. The authors do not routinely balloon mould through the fenestrated segment in order to preserve target vessel stent alignment. The majority of these endoleaks (29 of 35) had resolved without intervention by the 1‐month surveillance imaging. An adjusted primary technical success rate of 95·4 per cent (165 of 173) is more representative of data from other centres, which may have different intraoperative reporting practices. Alternatively, heterogeneity in intraoperative heparinization regimens and attitudes to time for stent‐graft conformation to occur, without balloon moulding, may account for the discrepancy in primary endoleak rates^9^.

Freedom from target vessel loss was 90·1 per cent at 5 years in this series. The attrition rate of target vessels is therefore surprisingly low even in the long term, and none of the target vessels that occluded resulted in serious clinical consequences for any patient. This is within the context of a robust surveillance programme which identifies and can act upon threats to target vessel patency. These results are in line with those of another long‐term study^4^ with median follow‐up of 67 months, confirming that target vessel loss does not appear to be a significant problem with fenestrated technology.

The present data appear to confirm the benefit of fenestrated EVAR technology for juxtarenal aneurysm repair in the long term. There were no aneurysm‐related deaths in longer‐term follow‐up and the overall survival was encouraging for a population of patients with aortic aneurysm. A significant number of complications were identified during postoperative surveillance; however, interventions to deal with these seemed effective. Secondary interventions performed after discharge were, in the main, endovascular procedures. The frequency of secondary intervention (37·2 per cent over 5 years) confirms the need for continued stent‐graft surveillance.

**Supporting information**

Additional supporting information may be found in the online version of this article:

**Fig. S1** Aneurysm diameter by calendar year (Word document)

**Fig. S2** Stent‐graft D1 diameter (neck diameter) by calendar year (Word document)

**Fig. S3** Freedom from mortality (all‐cause) for 10 years following fenestrated endovascular aneurysm repair (Word document)

**Fig. S4** Freedom from graft‐related endoleak following fenestrated endovascular aneurysm repair (Word document)

**Fig. S5** Freedom from abdominal aortic aneurysm growth (more than 5 mm) following fenestrated endovascular aneurysm repair (Word document)

**Fig. S6** Freedom from secondary intervention following fenestrated endovascular aneurysm repair (Word document)

**Fig. S7** Freedom from loss of any target vessel following fenestrated endovascular aneurysm repair (Word document)

**Fig. S8** Freedom from abdominal aneurysm growth (more than 5 mm) following fenestrated endovascular aneurysm repair in relation to number of fenestrations in stent‐graft (Word document)

**Fig. S9** Freedom from loss of a target vessel following fenestrated endovascular aneurysm repair in a single UK centre in relation to number of fenestrations in stent‐graft (Word document)

**Fig. S10** Freedom from mortality (all‐cause) following fenestrated endovascular aneurysm repair in relation to number of fenestrations in stent‐graft (Word document)

## Snapshot quiz 17/7

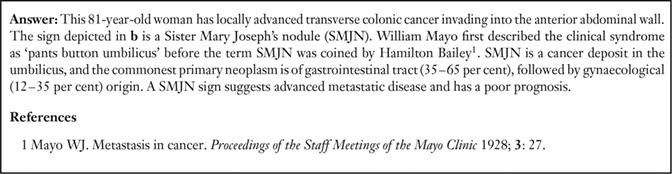

## Supporting information


**Fig. S1** Aneurysm diameter by calendar year. Median value (bold line), i.q.r. (box), and range (errors bars) excluding outliers (circles) are shown
**Fig. S2** Stent‐graft D1 diameter (neck diameter) by calendar year. Median value (bold line), i.q.r. (box), and range (errors bars) excluding outliers (circles) are shown
**Fig. S3** Freedom from mortality (all cause) for 10 years following fenestrated endovascular aneurysm repair, with 95 per cent confidence intervals
**Fig. S4** Freedom from graft‐related endoleak following fenestrated endovascular aneurysm repair, with 95 per cent confidence intervals
**Fig. S5** Freedom from abdominal aortic aneurysm (AAA) growth (more than 5 mm) following fenestrated endovascular aneurysm repair, with 95 per cent confidence intervals
**Fig. S6** Freedom from secondary intervention following fenestrated endovascular aneurysm repair, with 95 per cent confidence intervals
**Fig. S7** Freedom from loss of any target vessel following fenestrated endovascular aneurysm repair, with 95 per cent confidence intervals
**Fig. S8** Freedom from abdominal aneurysm (AAA) growth (more than 5 mm) following fenestrated endovascular aneurysm repair in relation to number of fenestrations in stent‐graft. P = 0·160 (log rank test)
**Fig. S9** Freedom from loss of a target vessel following fenestrated endovascular aneurysm repair in a single UK centre in relation to number of fenestrations in stent‐graft. P = 0·272 (log rank test)
**Fig. S10** Freedom from mortality (all cause) following fenestrated endovascular aneurysm repair in a single UK centre in relation to number of fenestrations in stent‐graft. P = 0·264 (log rank test)
