# Supplementary material for: Long‐term follow‐up of fenestrated endovascular repair for juxtarenal aortic aneurysm
Source: Br J Surg. 2017 Apr 12;104(8):1020–7. doi: 10.1002/bjs.10524 (PMC5485015; doi:10.1002/bjs.10524)
Supplement: Supplementary file 1 — Fig. S1 Aneurysm diameter by calendar year. Median value (bold line), i.q.r. (box), and range (errors bars) excluding outliers (circles) are shown Fig. S2 Stent‐graft D1 diameter (neck diameter) by calendar year. Median value (bold line), i.q.r. (box), and range (errors bars) excluding outliers (circles) are shown Fig. S3 Freedom from mortality (all cause) for 10 years following fenestrated endovascular aneurysm repair, with 95 per cent confidence intervals Fig. S4 Freedom from graft‐related endoleak following fenestrated endovascular aneurysm repair, with 95 per cent confidence intervals Fig. S5 Freedom from abdominal aortic aneurysm (AAA) growth (more than 5 mm) following fenestrated endovascular aneurysm repair, with 95 per cent confidence intervals Fig. S6 Freedom from secondary intervention following fenestrated endovascular aneurysm repair, with 95 per cent confidence intervals Fig. S7 Freedom from loss of any target vessel following fenestrated endovascular aneurysm repair, with 95 per cent confidence intervals Fig. S8 Freedom from abdominal aneurysm (AAA) growth (more than 5 mm) following fenestrated endovascular aneurysm repair in relation to number of fenestrations in stent‐graft. P = 0·160 (log rank test) Fig. S9 Freedom from loss of a target vessel following fenestrated endovascular aneurysm repair in a single UK centre in relation to number of fenestrations in stent‐graft. P = 0·272 (log rank test) Fig. S10 Freedom from mortality (all cause) following fenestrated endovascular aneurysm repair in a single UK centre in relation to number of fenestrations in stent‐graft. P = 0·264 (log rank test) [file BJS-104-1020-s001.docx]

**BJS10524**

**Long-term follow-up of fenestrated endovascular repair for juxta-renal aortic aneurysm**

I. N. Roy, A. M. Millen, S. M. Jones, S. R. Vallabhaneni, J. R. H. Scurr, R. G. McWilliams, J. A. Brennan and R. K. Fisher


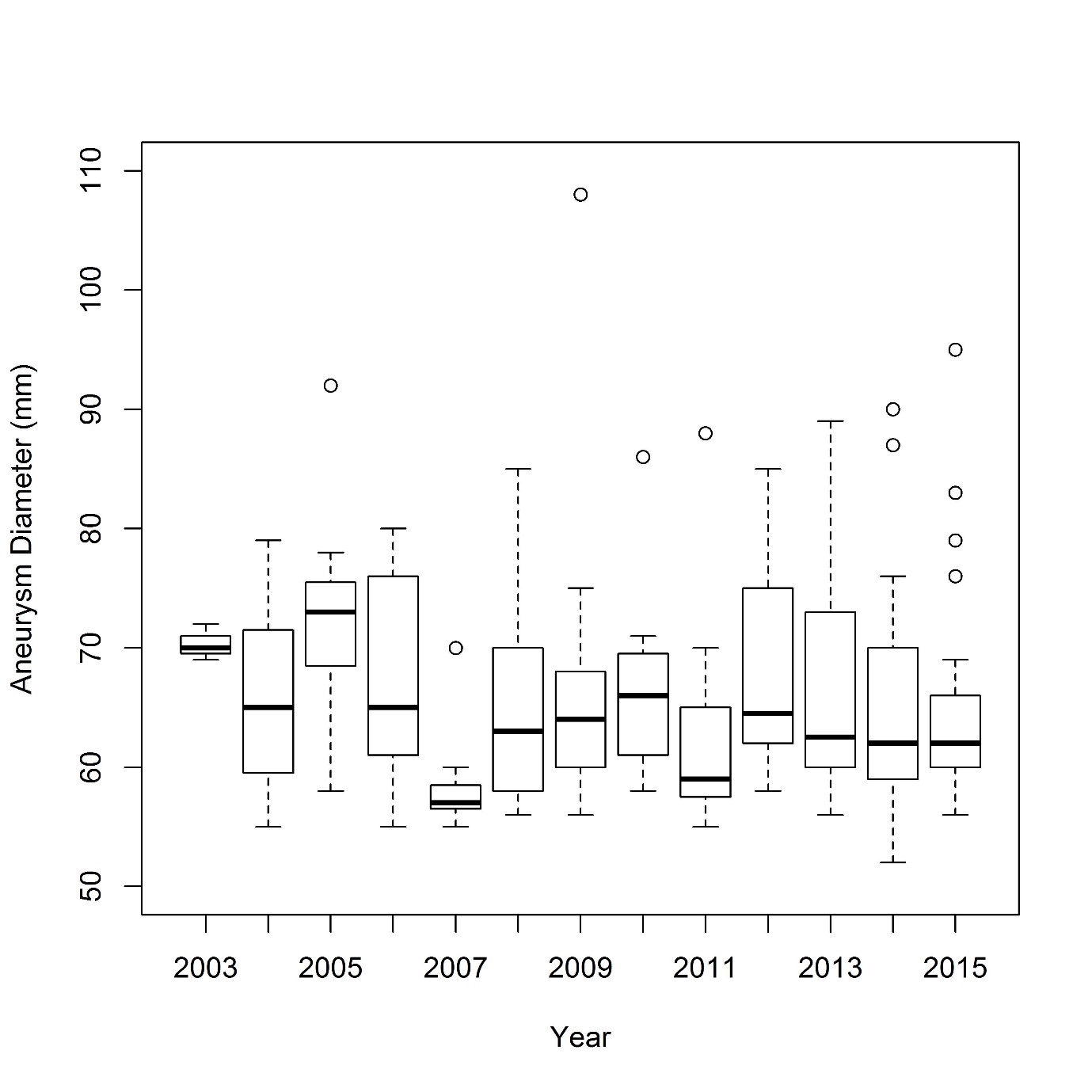


**Fig. S1** Aneurysm diameter by calendar year. Median value (bold line), i.q.r. (box), and range (errors bars) excluding outliers (circles) are shown

**
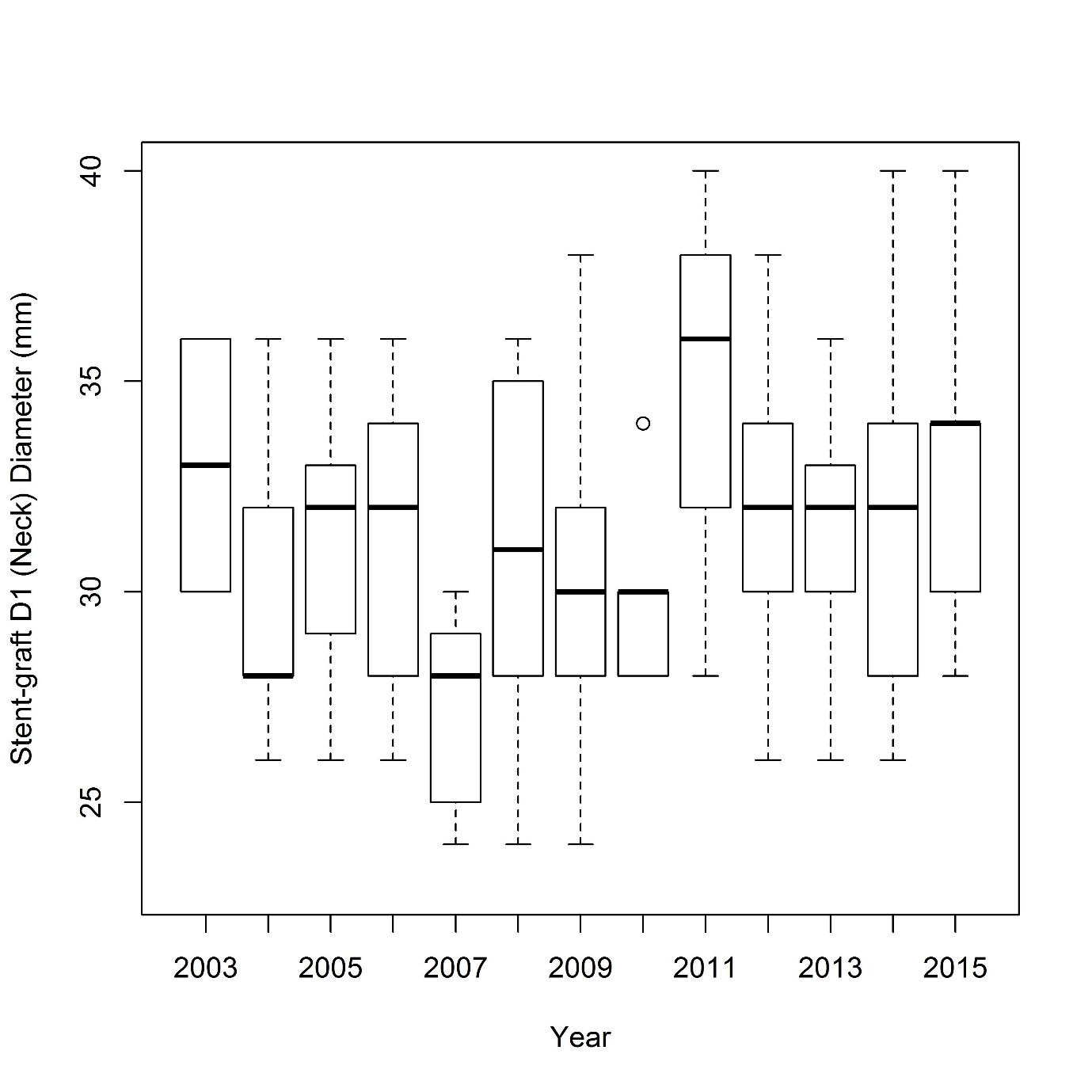
**

**Fig. S2** Stent-graft D1 diameter (neck diameter) by calendar year. Median value (bold line), i.q.r. (box), and range (errors bars) excluding outliers (circles) are shown

**
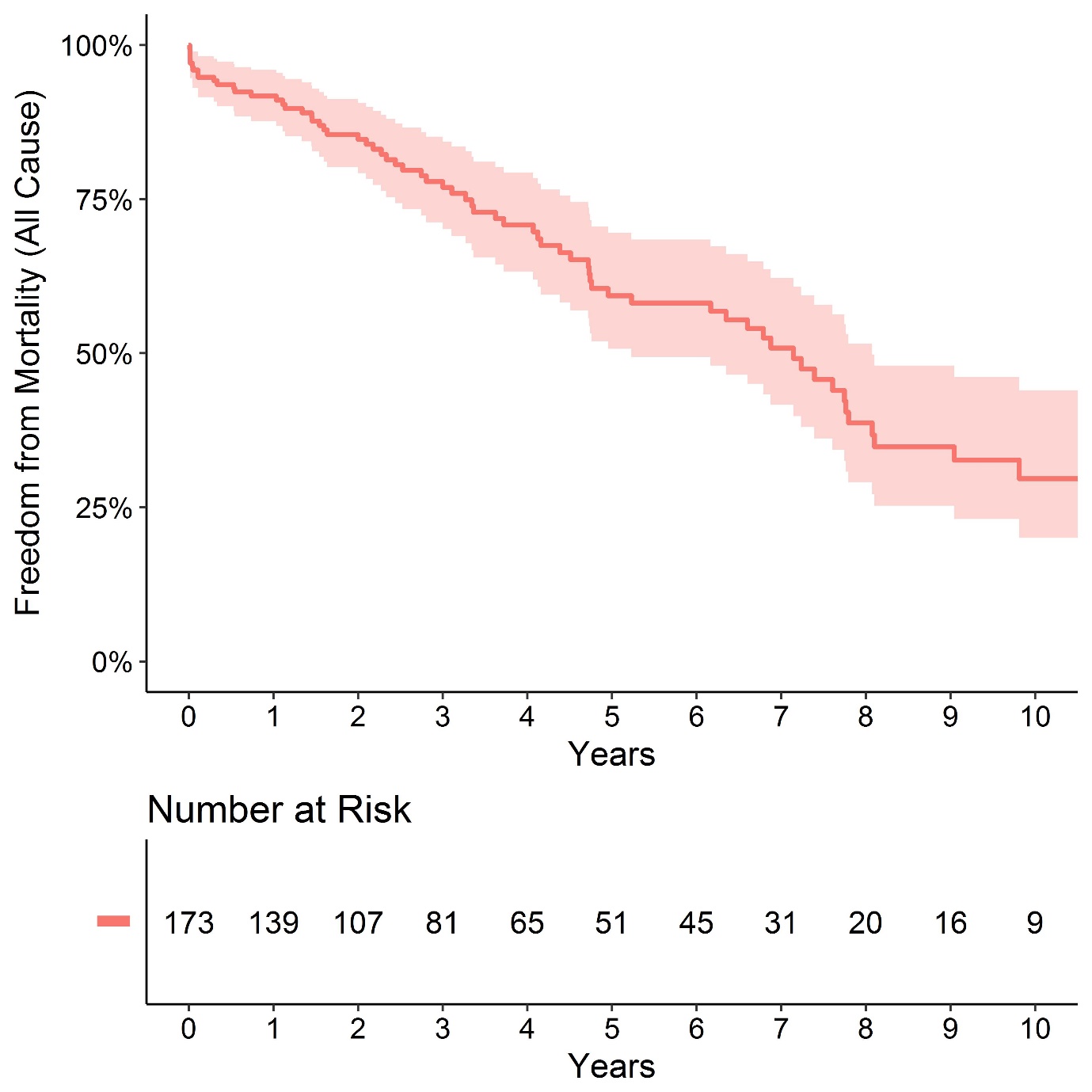
**

**Fig. S3** Freedom from mortality (all cause) for 10 years following fenestrated endovascular aneurysm repair, with 95 per cent confidence intervals

**
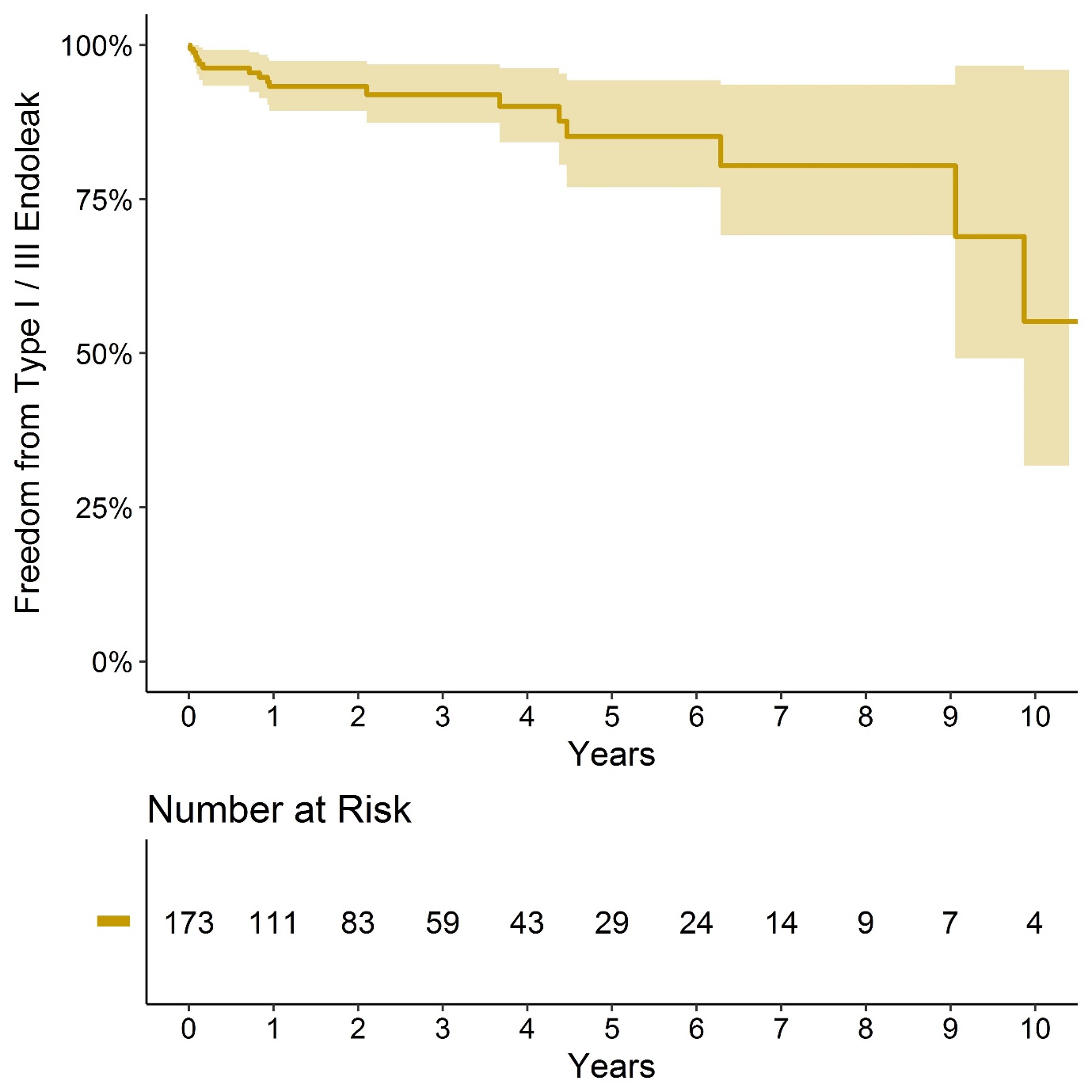
**

**Fig. S4** Freedom from graft-related endoleak following fenestrated endovascular aneurysm repair, with 95 per cent confidence intervals

**
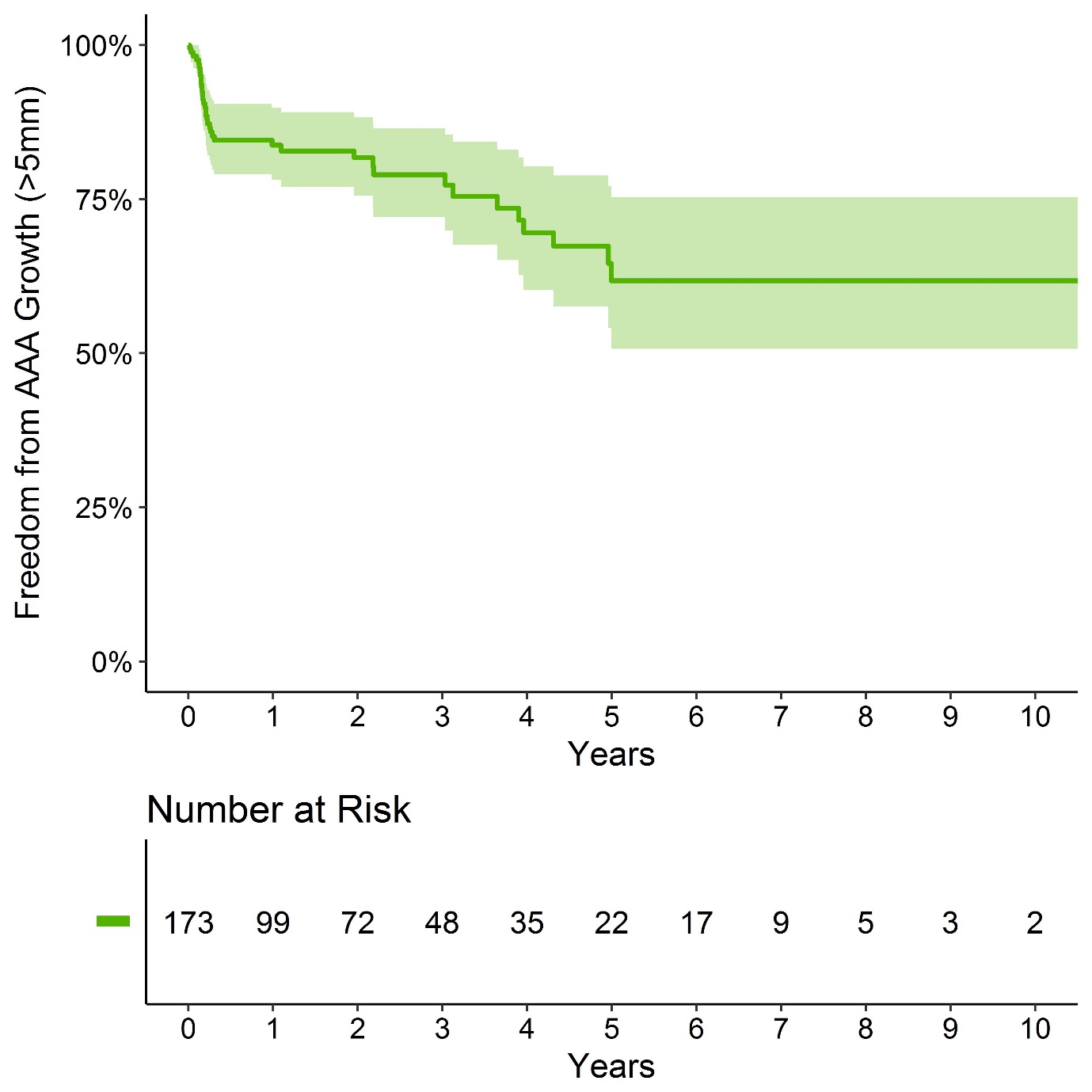
**

**Fig. S5** Freedom from abdominal aortic aneurysm (AAA) growth (more than 5 mm) following fenestrated endovascular aneurysm repair, with 95 per cent confidence intervals

**
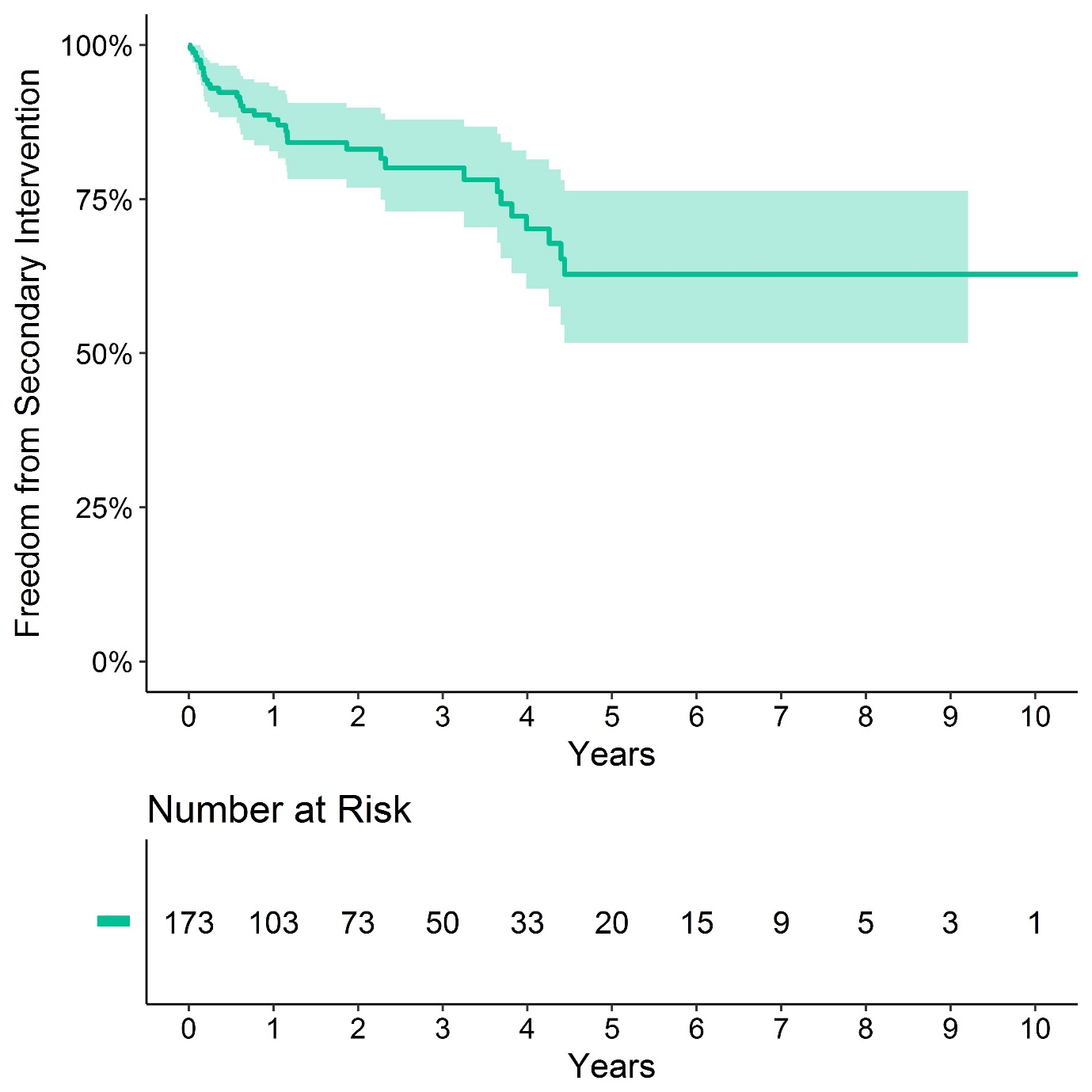
**

**Fig. S6** Freedom from secondary intervention following fenestrated endovascular aneurysm repair, with 95 per cent confidence intervals

**
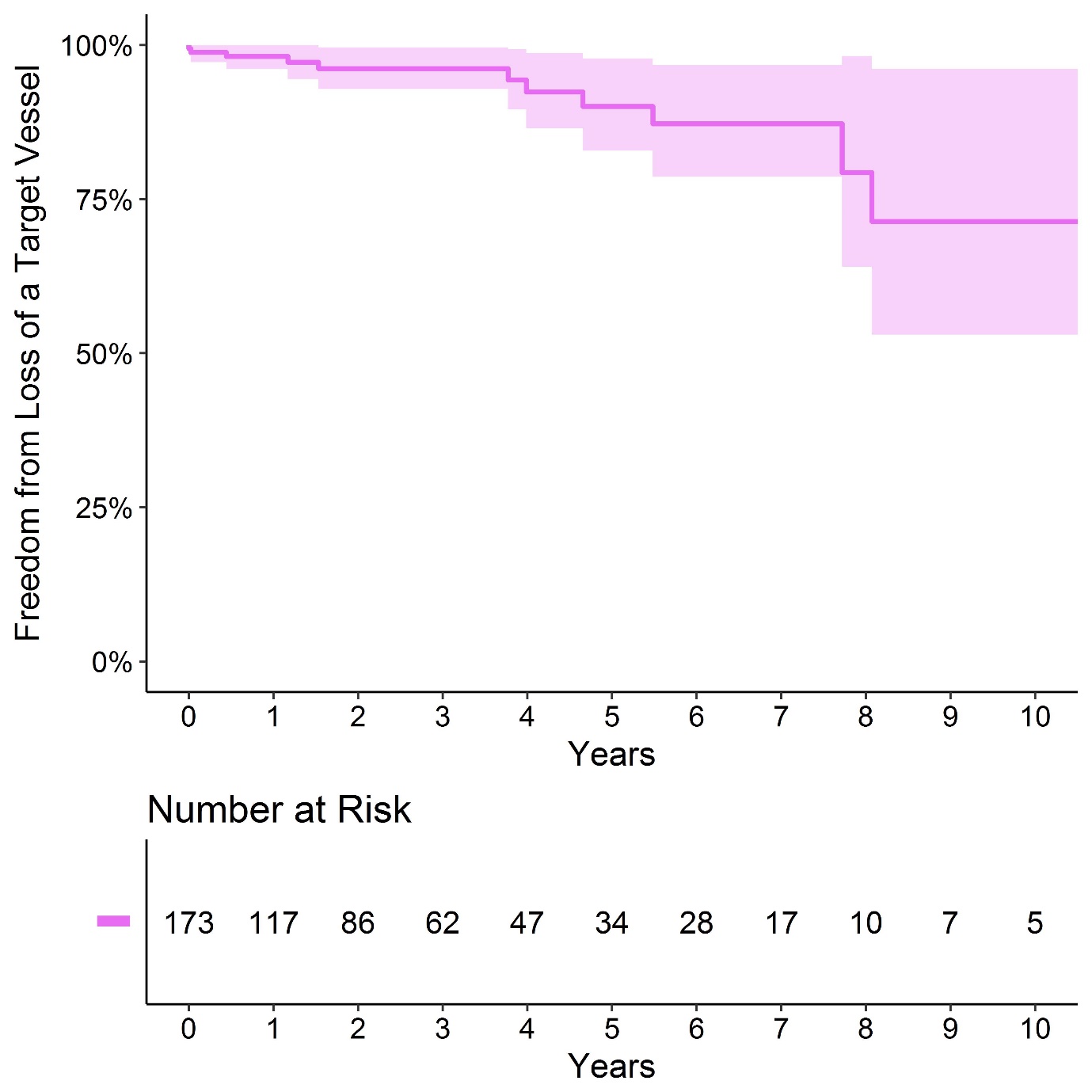
**

**Fig. S7** Freedom from loss of any target vessel following fenestrated endovascular aneurysm repair, with 95 per cent confidence intervals

**
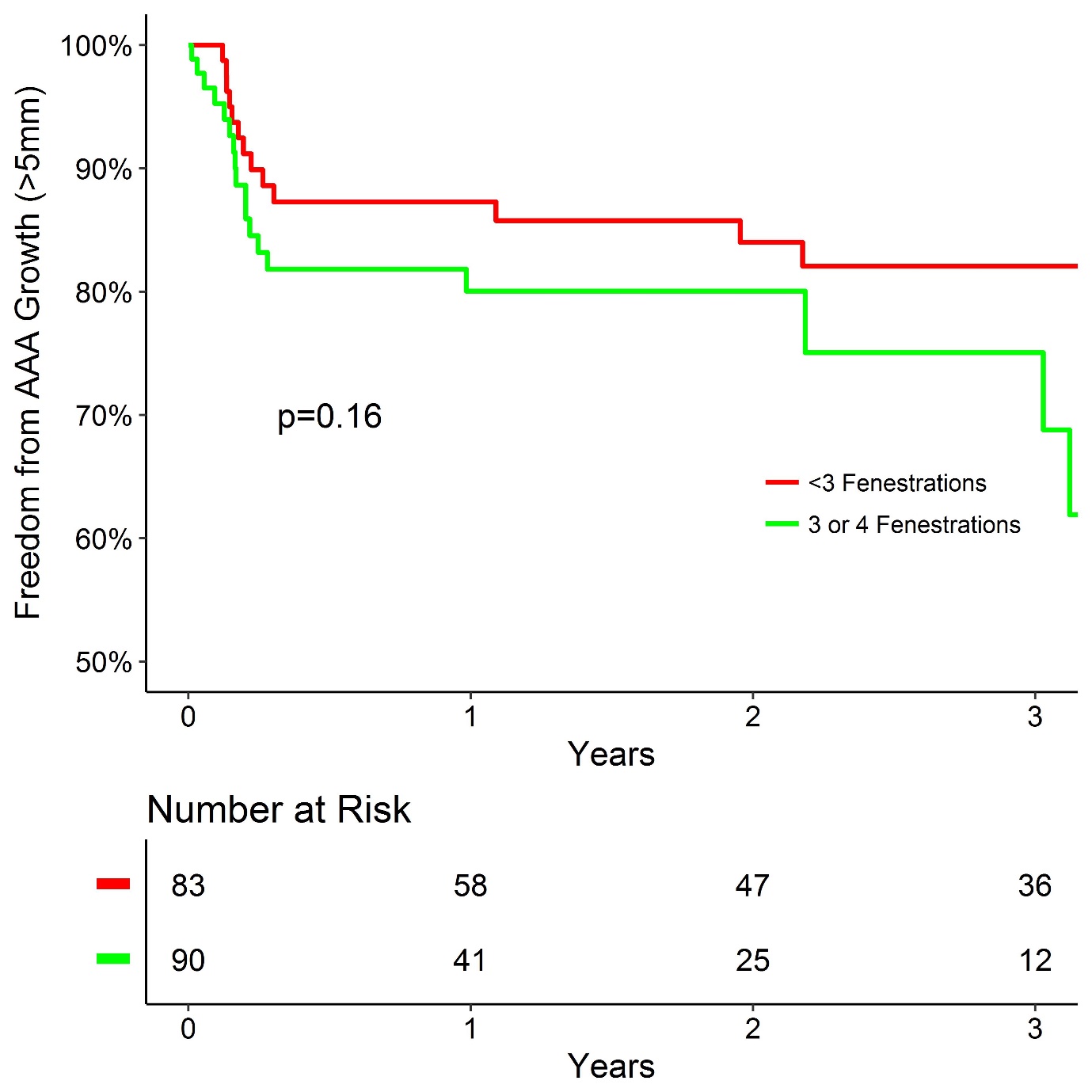
**

**Fig. S8** Freedom from abdominal aneurysm (AAA) growth (more than 5 mm) following fenestrated endovascular aneurysm repair in relation to number of fenestrations in stent-graft. *P* = 0.160 (log rank test)

**
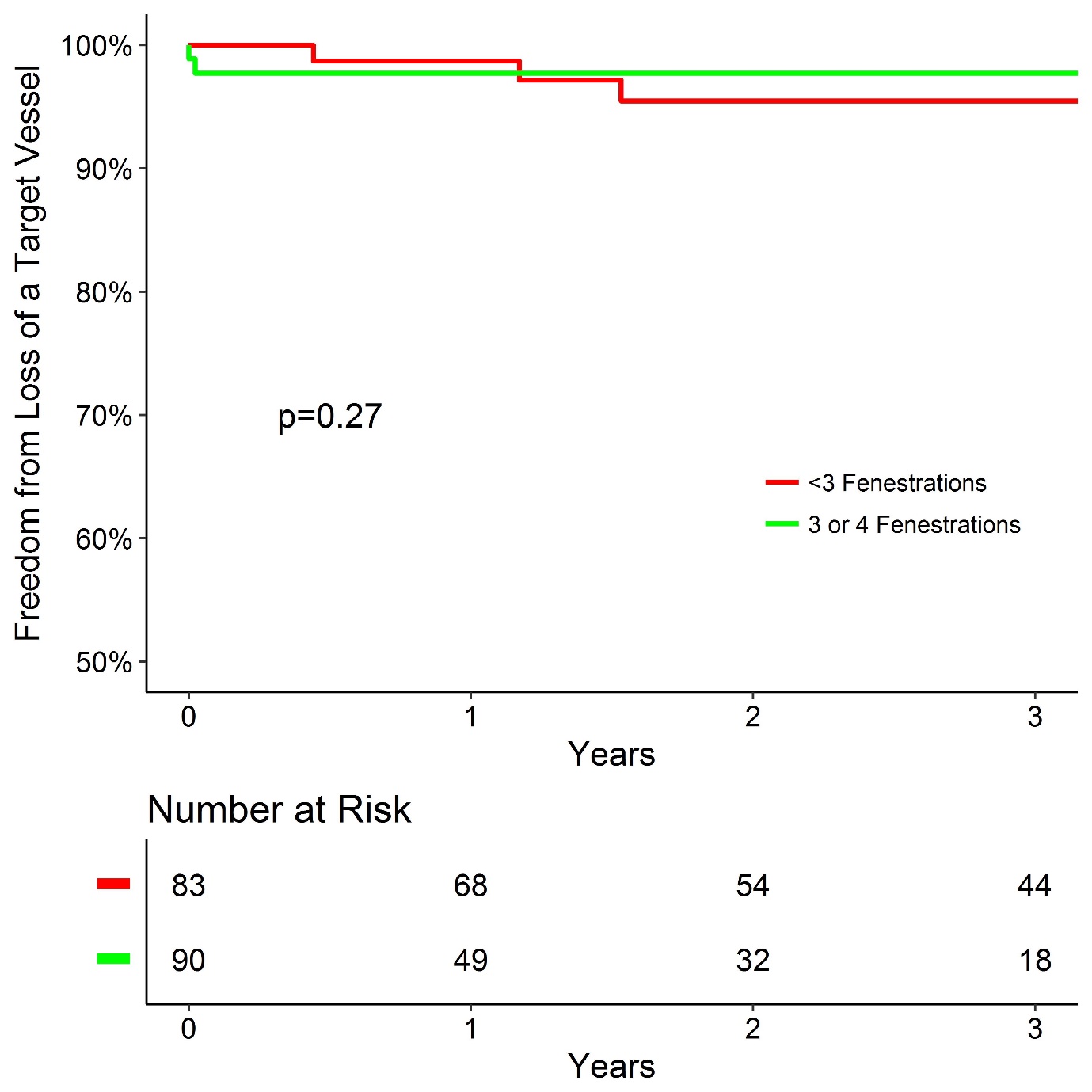
**

**Fig. S9** Freedom from loss of a target vessel following fenestrated endovascular aneurysm repair in a single UK centre in relation to number of fenestrations in stent-graft. *P* = 0.272 (log rank test)

**
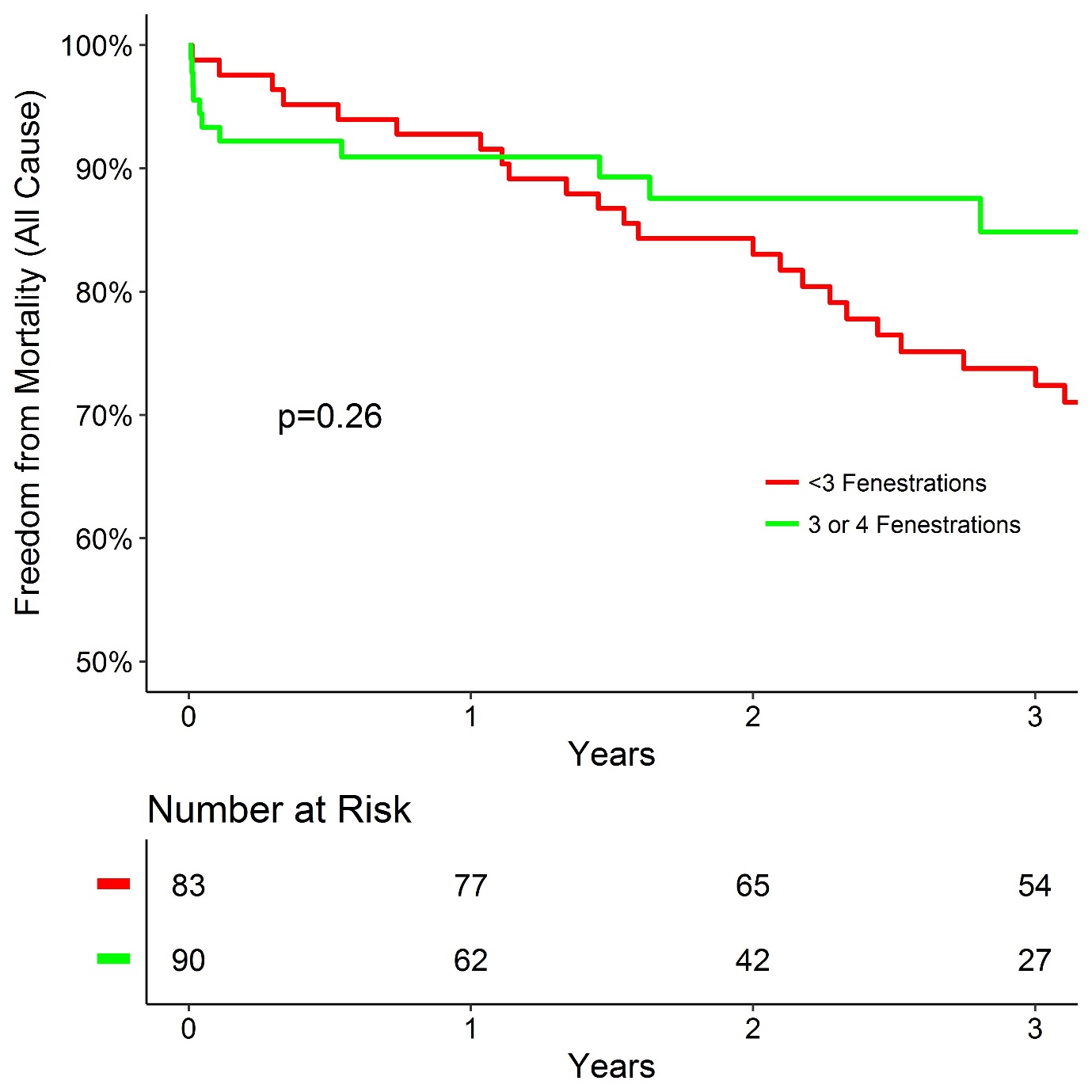
Fig. S10** Freedom from mortality (all cause) following fenestrated endovascular aneurysm repair in a single UK centre in relation to number of fenestrations in stent-graft. *P* = 0.264 (log rank test)
